# CD36 promotes vasculogenic mimicry in melanoma by mediating adhesion to the extracellular matrix

**DOI:** 10.1186/s12885-021-08482-4

**Published:** 2021-07-02

**Authors:** Carmela Martini, Mark DeNichilo, Danielle P. King, Michaelia P. Cockshell, Brenton Ebert, Brian Dale, Lisa M. Ebert, Anthony Woods, Claudine S. Bonder

**Affiliations:** 1 Centre for Cancer Biology, University of South Australia and SA Pathology, Adelaide, South Australia Australia; 2 Clinical & Health Sciences, University of South Australia, Adelaide, South Australia Australia; 3 Adelaide Medical School Faculty of Health and Medical Sciences, The University of Adelaide, Adelaide, South Australia Australia

**Keywords:** CD36, Vasculogenic mimicry, Melanoma, Tumor microenvironment, Thrombospondin, Laminin, Integrin

## Abstract

**Background:**

The formation of blood vessels within solid tumors directly contributes to cancer growth and metastasis. Until recently, tumor vasculature was thought to occur exclusively via endothelial cell (EC) lined structures (i.e. angiogenesis), but a second source of tumor vasculature arises from the cancer cells themselves, a process known as vasculogenic mimicry (VM). While it is generally understood that the function of VM vessels is the same as that of EC-lined vessels (i.e. to supply oxygen and nutrients to the proliferating cancer cells), the molecular mechanisms underpinning VM are yet to be fully elucidated.

**Methods:**

Human VM-competent melanoma cell lines were examined for their VM potential using the in vitro angiogenesis assays (Matrigel), together with inhibition studies using small interfering RNA and blocking monoclonal antibodies. Invasion assays and adhesion assays were used to examine cancer cell function.

**Results:**

Herein we demonstrate that CD36, a cell surface glycoprotein known to promote angiogenesis by ECs, also supports VM formation by human melanoma cancer cells. In silico analysis of *CD36* expression within the melanoma cohort of The Cancer Genome Atlas suggests that melanoma patients with high expression of *CD36* have a poorer clinical outcome. Using in vitro ‘angiogenesis’ assays and CD36-knockdown approaches, we reveal that CD36 supports VM formation by human melanoma cells as well as adhesion to, and invasion through, a cancer derived extracellular matrix substrate. Interestingly, thrombospondin-1 (TSP-1), a ligand for CD36 on ECs that inhibits angiogenesis, has no effect on VM formation. Further investigation revealed a role for laminin, but not collagen or fibronectin, as ligands for CD36 expressing melanoma cells.

**Conclusions:**

Taken together, this study suggests that CD36 is a novel regulator of VM by melanoma cancer cells that is facilitated, at least in part, via integrin-α_3_ and laminin. Unlike angiogenesis, VM is not perturbed by the presence of TSP-1, thus providing new information on differences between these two processes of tumor vascularization which may be exploited to combat cancer progression.

## Background

Angiogenesis is the most common mechanism by which tumors become vascularized and metastasize. However, the growing mass can also utilise other processes to form vascular networks, such as vasculogenic mimicry (VM). First identified in aggressive uveal melanoma cells, VM has since been seen in several aggressive malignancies such as glioblastoma [1–3], colorectal cancer [4, 5], breast cancer [6, 7], ovarian cancer [8, 9], pancreatic cancer [10] and prostate cancer [11]. VM occurs when highly aggressive malignant cells undergo cellular phenotypic changes to resemble endothelial cells (ECs) which form the inner lining of all vasculature. While these malignant cells display EC markers, i.e. vascular endothelial (VE)-cadherin, ephrin receptor A2 (EphA2) and E-selectin, and like ECs can secrete many basement membrane relevant proteins, they do however have little to no expression of EC-specific proteins such as CD31 (PECAM-1) and TIE-2 [12, 13]. Histological staining with the periodic acid-Schiff stain (PAS, recognizing basement membrane proteins) used in conjunction with an antibody to CD31 is the universally accepted protocol to distinguish VM structures as PAS-positive/CD31-negative from the EC-lined PAS-positive/CD31-positive vasculature [14–16].

The aggressive nature of VM is supported by evidence of these structures anastomosing with traditional EC-lined tumor vasculature [17]; and in doing so, it further enables the solid tumor mass to gain access to oxygen and nutrients for growth [18]. VM content in tumors is undeniably an indicator of poor patient prognosis, including metastatic disease and overall survival [16, 19]. Meta-analyses of different cancer types show that patients with tumors that are high in VM content have a significantly lower 5-year overall survival rate when compared to those with little/no VM content [20]. Despite the similarities between angiogenesis and VM, current anti-angiogenic approaches fail to target VM vascular structures and indeed the use of antiangiogenic therapy (bevacizumab) has been shown to increase VM in ovarian and breast cancer [21, 22]. Anti-VM therapies have had limited success, with currently only one drug, CVM-1118 (NCT03582618), in Phase 2 open label clinical trial [23, 24]. Hence, understanding the mechanisms involved in VM formation is clearly clinically important.

CD36 is an 88 kDa cell surface glycoprotein well documented as a scavenger receptor on many cell types, including platelets, phagocytic cells, adipocytes, erythrocytes, specialized epithelia, myocytes and microvascular endothelium (reviewed in [25]). The functional diversity of CD36 is underpinned by its binding to distinct ligands including long chain fatty acids, phospholipids, oxidized low-density lipoprotein (oxLDL), amyloid proteins, advanced glycation end products and thrombospondin (TSP) [25]. In tumor tissues, CD36 is expressed by cancer cells, ECs, stromal cells and immune cells [26] with numerous studies supporting the notion that CD36 participates in the progression of cancer [27–29]. More specifically, retrospective analyses suggest that elevated levels of *CD36* correlate with poor prognosis in patients with glioma, cervical cancer, ovarian cancer, lung cancer, squamous-cell carcinoma, bladder cancer, and luminal A breast cancer [27–30]. Consistent with this, overexpression studies have shown CD36 to increase the migration and invasion of cervical cancer cells in vitro, and its knockdown inhibited their metastatic potential [30]. Pascual and colleagues further demonstrated that short hairpin RNA-mediated depletion of CD36 significantly reduced metastases in models of melanoma (501mel) and breast cancer (MCF-7) in vivo [29]. Hypoxia is a well-known initiator of tumor vascularization and hypoxia has also been documented to elevate CD36 expression on microvascular ECs (MVECs) [31]. Taken together, these findings begin to reveal an important inter-relationship between cancer cells, tumor ECs and CD36. Interestingly, the CD36 ligand TSP-1 has been identified as a potent inhibitor of angiogenesis as it renders ECs non-responsive to pro-angiogenic stimuli such as vascular endothelial growth factor (VEGF) via changes in intracellular signaling pathways [32, 33] and its promotion of apoptosis [34]. Elevated levels of TSP-1 have indicated some suppressive control of tumor growth and cancer metastasis [35], likely via inhibition of tumor vasculature.

On the whole, the expression of CD36 has poor implications in cancer, and while CD36 has gained significant attention in recent years, its role in tumor vasculature and cancer progression is still not fully understood. The aim of the present study is to investigate the currently unknown role of CD36 in VM by melanoma cells.

## Methods

### Bioinformatics analysis of publicly available datasets

To analyze The Cancer Genome Atlas (TCGA) data, RNA sequencing (RNA-seqV2) and clinical (Biotab) data were downloaded from the Data Portal at http://cancergenome.nih.gov/. Data were analyzed in Bioconductor, using the edgeR package to perform differential gene expression analysis. A Kaplan Meier plot was generated to compare the overall survival between patients expressing the top 10% of *CD36* against the patients expressing the bottom 10% of *CD36.*

### Cell culture

Human melanoma cell lines C32 and SK-MEL-28 were gifted from G McArthur (Peter MacCallum Cancer Centre, Melbourne, Vic, AUS) and cultured in RPMI 1640 (Gibco by Life Technologies, California, USA) containing 10% fetal bovine serum (FBS) (Hyclone, GE Healthcare Sciences, Utah, USA) and 1% Glutamax 1x (Gibco) and cultured under standard conditions (37 °C, 5% CO_2_). The isolation of endothelial colony-forming cells (ECFCs) from healthy human peripheral blood, as previously described [36], was approved by the human ethics committees of the University of South Australia (UniSA HREC #201187). Briefly, collagen I-coated plates were seeded with lymphoprep enriched mononuclear cells and cultured in EGM-2 media (Lonza) containing 20% Embryonic Stem cell screened fetal bovine serum (Hyclone, GE Healthcare, Chicago, IL, USA) until colony formation at ~ 14 days culture after which time the cells were passaged and cultured for no more than 9 passages. Human lung microvascular endothelial cells (HMVEC) were purchased from the Lonza (Basel, Switzerland) and cultured in Lonza EBM-2 basal medium to low passage number.

### Flow cytometry

To determine cell surface expression of CD36, cells were labelled with anti-human CD36 antibody (FA6–152, Stem Cell Technologies, Vancouver, Canada) or isotype control (IgG_1_ κ) at a concentration of 10 μg/ml in RPMI1640 media (Sigma-Aldrich, Merck, Sydney Australia) containing 10% FBS. Cells were then washed and stained with a secondary antibody, goat anti-mouse IgG H&L Dylight 650 (Clone ab96882, Abcam, Cambridge, United Kingdom) at a concentration of 5 μg/ml. Similarly, cells were labelled with directly conjugated antibodies within panels containing one or more of the following; anti-CD144 (VE-cadherin)-FITC, anti-CD31(PECAM)-PE, anti-Tie-2-Alexa Fluor 647, anti-CD146 (MCAM)-PE (all BD Bioscience) and anti-CD309 (VEGFR2)-Alexa Fluor 647 (Biolegend), with non-specific isotype control antibodies, IgG1-FITC, IgG1-PE and IgG1-Alexa Fluor 647 (all BD Bioscience). Cell viability was determined via 7AAD (BD Biosciences) staining at a 1:20 dilution. Samples were processed using a BD Accuri C6 flow cytometer (BD, Becton, Dickinson and Company, New Jersey, USA) and data analyzed using FCS Express 4 Flow Research Edition (De Novo software, Los Angeles, USA).

### CD36 knockdown using siRNA

CD36 targeting small interfering RNAs (siRNAs) Trilencer-27 Human siRNA duplexes at 20 μM were purchased (Origene, Maryland, USA) with three CD36 targeting siRNAs (duplex sequences: SR319610**A** rCrArArCrCrUrArUrUrGrGrUrCrArArGrCrCrArUrCrArGAA, SR319610**B** rGrGrCrCrUrGrArUrArGrArArArUrGrArUrCrUrUrArCrUCA and SR319610**C** rGrGrArUrUrArArArCrCrCrArArArUrGrArArGrArArGrAAC) plus a non-targeting scrambled (SCR) siRNA used as a negative control. Transfection was performed using Lipofectamine RNAiMAX (Invitrogen by Life Technologies, California, USA) as per the manufacturer’s instructions with the knockdown maximized at 5 nM and validated by flow cytometry.

### Vasculogenic mimicry (VM) assays

VM assays were performed on Geltrex LDEV-Free Reduced Growth Factor Basement Membrane Matrix (Life Technologies, California, USA) with 10 μl coating each of a 15 well μ-Angiogenesis culture slides (Ibidi, Martinsreid, Germany) and allowed to solidify over 30 min at 37 °C. Human melanoma cancer cells were then seeded at 2 × 10^4^ cells/well for C32 cells and 5 × 10^3^ cells/well for SK-MEL-28 cells and after 6 h incubation at 37 °C, images of each well were obtained using the EVOS XL Core Imaging System (Thermo Fisher Scientific) with brightness of the images adjusted equally across groups to enhance image contrast for ease of readers. VM structures were defined as multi-cellular arrangements forming vessel-like structures from one branch point to another [16] and VM in each well was counted in blinded manner. A minimum of three independent experiments were performed. Similar experiments included cells without or with CD36-targeting siRNA knockdown or those pre-treated with TSP-1 (150 or 1500 ng/ml), an anti-CD36 blocking antibody (10 μg/ml, FA6–152) or an isotype control (IgG_1_) for 30 min at 4 °C prior to seeding.

### Angiogenesis assays

Human lung microvascular endothelial cells (HMVEC) were serum starved overnight in EBM-2 basal medium (Lonza, Basel, Switzerland) at 37 °C prior to seeding onto Geltrex at a concentration of 2 × 10^4^ cells/well within a 15 well μ-Angiogenesis slide with or without the addition of TSP-1 (1500 ng/ml) in EBM-2 basal medium containing EGM-2 MV SingleQuot Kit supplements and growth factors (Lonza, Basel, Switzerland). After 6 h, images of the angiogenesis formation were obtained using the EVOS XL Cell Imaging System. Angiogenesis formation was determined by blindly counting the vessel-like structures formed within each well with the use of ImageJ software.

### MTS survival assay

HMVEC were seeded into 96-well flat bottom plates at a concentration of 2 × 10^4^ cells/well in EBM-2 basal medium containing EGM-2 MV SingleQuot Kit supplements and growth factors. Cells were incubated at 37 °C for 5 h, washed in 1x PBS, serum starved in EBM-2 basal medium overnight at 37 °C and then treated without or with TSP-1 (1500 ng/ml) for 6 h and 24 h at 37 °C. MTS (3-(4,5-dimethylthiazol-2-yl)-5-(3-carboxymethoxyphenyl)-2-(4-sulfophenyl)-2H-tetrazolium; Life Technologies, California, USA) was then added to each well at a concentration of 0.5 mg/ml for 2 h at 37 °C. Viable cell density was determined by measuring absorbance at 490 nm on the Epoch microplate spectrophotometer (Biotek, USA).

### Inverse invasion assays

Using previously described methods [37], growth factor-reduced Matrigel diluted 1:1 in cold PBS was loaded into Transwells (Corning Inc., NY, USA) to set prior to being inverted and C32 cells (2 × 10^5^/ml ± CD36 knockdown via siRNA) were seeded onto the underside of the membrane. After four hours, the unbound cells were washed away and Transwells immersed right-way up in serum-free HUVEC media ±10% FBS to the upper chamber and cells allowed to migrate upward into the Matrigel for two days. Transwells were fixed with paraformaldehyde for 30 min, washed with PBS, RNAse-treated (100 μg/ml, Thermo Fisher) for 30 min, washed with PBS, and stained with propidium iodide (0.05 mg/ml, Thermo Fisher). Starting at the membrane, Transwells were imaged at 10 μm fixed intervals in a direction towards the chemoattractant using Zeiss LSM 700 confocal microscope with a 20x objective and z-stack setting (Carl Zeiss AG, Oberkochen, Germany). From three fields of view per slice, images were quantified using ImageJ software, through threshold adjustment and counting particles (cells), these were then averaged.

### Adhesion assays

C32 melanoma cells (1.5 × 10^4^ cells) were seeded into wells of a flat bottom 96-well plate preloaded overnight at 4 °C with 10–50 μg/ml collagen I (Becton, Dickinson and Company, New Jersey, USA), collagen IV (Sigma-Aldrich, Missouri, United States), laminin (Roche, Basel, Switzerland), fibronectin (Roche, Basel, Switzerland) or Geltrex. Cells that had undergone a 72-h CD36-targeting siRNA knockdown were harvested and resuspended in 1 mg/ml of bovine serum albumin (BSA) (Sigma Aldrich, Missouri, USA) in RPMI 1640 and seeded into the extracellular matrix (ECM) component containing wells and incubated at 37 °C for 90 min. Rose Bengal (Sigma Aldrich, Missouri, USA) diluted in warm 1x PBS to a concentration of 0.05%, was then added to the cells for 10 min at RT prior to several washes with PBS. A 1:1 solution of methanol and PBS was then added to the cells for 10 min prior to the absorbance reading via spectrophotometry at 562 nm FLUOstar Omega plate reader (BMG LABTECH GmbH, Offenberg Germany).

Functional blocking studies using the mouse anti-integrin-α_3_ mAb (Clone P1B5; Sigma Aldrich, Missouri, USA) were performed by pre-incubating C32 melanoma cells at 37 °C with anti-integrin-α_3_ mAb at 50 μg/mL for 30 min prior to cell attachment to wells coated with human laminin at 50 μg/ml as described above. Images of each well were obtained using the EVOS XL Core Imaging System.

### Immunocytochemistry

Circular glass coverslips (Menzel Glaser, Germany) were placed into a 24-well plate (Nunc) and coated overnight at 4 °C with EHS-derived mouse laminin (Roche) at 20 μg/mL in PBS. C32 melanoma cells were harvested and resuspended in RPMI 1640 medium containing BSA at 1 mg/mL; cells (5 × 10^4^ per well) were allowed to adhere and spread for 60 min at 37 °C, and nonadherent cells removed by gentle washing. Cells were then fixed with 4% paraformaldehyde/PBS for 10 min at room temperature. After washing with PBS, cells were permeated with 0.25% Triton X-100 (Sigma-Aldrich) in PBS for 10 min. Non-specific binding sites were blocked for 90 min using 3% BSA in PBS containing a 1:10 dilution of nonimmune goat serum. Cells were incubated overnight at 4 °C with the primary antibodies [mouse anti-human integrin-α_3_ at 1 μg/mL (clone P1B5, Merck Australia); rabbit anti-human paxillin at 0.5 μg/mL (sc-5574, Santa Cruz Biotechnology), mouse IgG1 isotype control at 1 μg/mL (BD Bioscience); and purified rabbit IgG at 0.5 μg/mL (Sigma-Aldrich)] in PBS containing BSA at 1 mg/mL and then counterstained with a 1:1000 dilution of Alexa Fluor 488–conjugated goat anti-rabbit and Alexa Fluor 555–conjugated goat anti-mouse IgG (Life Technologies) as well as a 1:3000 dilution of DAPI nuclear stain (Sigma-Aldrich) in PBS containing 1 mg/mL BSA. Coverslips were mounted onto glass slides using Fluoromount (Sigma-Aldrich), and images were captured using a 63x objective fluorescence confocal photomicroscope (LSM800, Carl Zeiss Microscopy, Jena, Germany).

### Statistical analysis

GraphPad PRISM software (San Diego, CA, USA) was used to perform statistical analyses and significance via a Mann Whitney U test or one-way ANOVA. In all comparisons, *p* < 0.05 was considered statistically significant.

## Results

### High *CD36* gene expression suggests poor clinical outcome for patients with melanoma

**Fig. 1:**
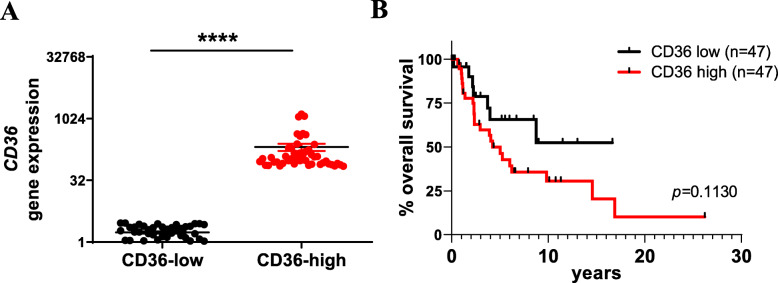
Association of clinical outcome in melanoma patients with high CD36 expression. **(A)** Patient data accessed from the TCGA-SKCM project (*n* = 470) was categorized into the top and bottom 10% of *CD36* gene expression (CD36-high (red) and CD36-low (black), respectively), ****p < 0.0001, unpaired t-test. **(B)** From the data in A, a Kaplan-Meier analysis was conducted between the CD36-high and CD36-low populations (n = 47 per group)

Data obtained from The Cancer Genome Atlas (TCGA) was used to determine the significance of *CD36* gene expression in the survival of patients with melanoma (TCGA-SKCM project, *n* = 470). Patient data was categorized into samples within the top 10% of *CD36* gene expression (CD36-high, n = 47) and those within the bottom 10% of *CD36* gene expression (CD36-low, n = 47) (Fig. 1A). Analysis of these two cohorts showed a clear trend of decreased overall survival in patients who have high *CD36* gene expression, compared to those with low *CD36* expression, although this difference was not statistically significant (Fig. 1B).

### Melanoma cells perform vasculogenic mimicry which is, in part, mediated by CD36

**Fig. 2:**
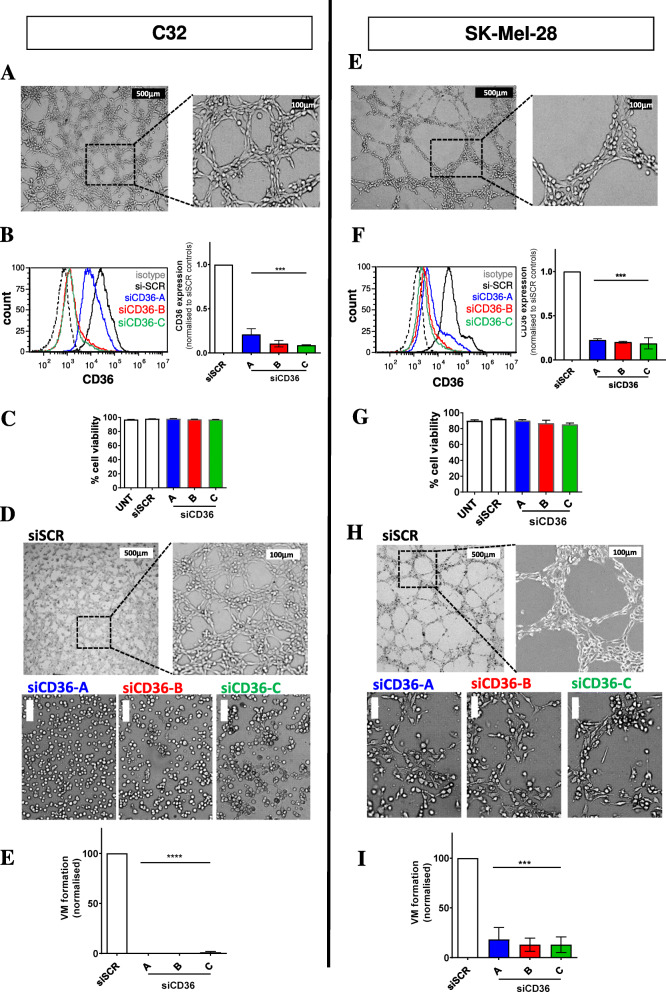
CD36 inhibition by siRNA attenuates VM by melanoma cells in vitro*.* **(A, E)** Representative images of C32 and SK-Mel-28 human melanoma cells undergoing VM. For each cell line, the left image depicts VM with low magnification (scale bar = 500 μm) and the right image is a zoomed in view of the same well using higher magnification (scale bar = 100 μm). **(B, F)** Left panels, flow cytometric histograms of CD36 expression on C32 and SK-Mel-28 cells with isotype control (dotted line), CD36 (solid black line), and siCD36 knockdown cells (A, blue line; B, red line and C, green line). Right panels, bar graphs of flow data quantified from experimental repeats, siCD36 (constructs A-C) normalized to scrambled siRNA (siSCR) controls. Data are expressed as mean ± SEM from *n* = 3 experiments.***p < 0.001 vs siSCR control, one-way ANOVA. **(C, G)** Cell viability without and with CD36 knockdown. Data are expressed as mean ± SEM from *n* = 3 experiments. **(D, H)** Top panels are representative images of C32 and SK-Mel-28 melanoma cells undergoing VM without or with siCD36 knockdown. For each cell line, the left image depicts VM with low magnification (scale bar = 500 μm) and the right image is a zoomed in view of the same well using higher magnification (scale bar = 100 μm). Lower panels illustrate the VM formation following CD36 knockdown for constructs RNAi A-C. (scale bar = 100 μm) **(E, I)** Quantitation of VM formation by the cancer cells, normalized to si-SCR control within each experiment. Data are expressed as mean ± SEM from *n* = 3 experiments. ***p < 0.001 vs si-SCR control, one-way ANOVA

To determine whether CD36 expression contributes to VM in melanoma, in vitro VM assays were performed using two human melanoma cells lines, C32 and SK-MEL-28 without and with CD36 knockdown. Figure 2A and E confirm the VM capability of both C32 and SK-MEL-28 cell lines in vitro with Fig. 2B and F demonstrating the ability of three different CD36-targeting siRNA constructs to consistently knockdown CD36 protein expression with an efficacy of up to 90%. Having first confirmed that knockdown of CD36 did not compromise the viability of the cells (Fig. 2C and G), we went on to examine the contribution of CD36 to VM formation. After 72 h of CD36 knockdown, VM formation was significantly decreased in both the C32 (Fig. 2D and E) and SK-MEL-28 (Fig. 2H and I) cell lines, resulting in fragmented networks.

**Fig. 3:**
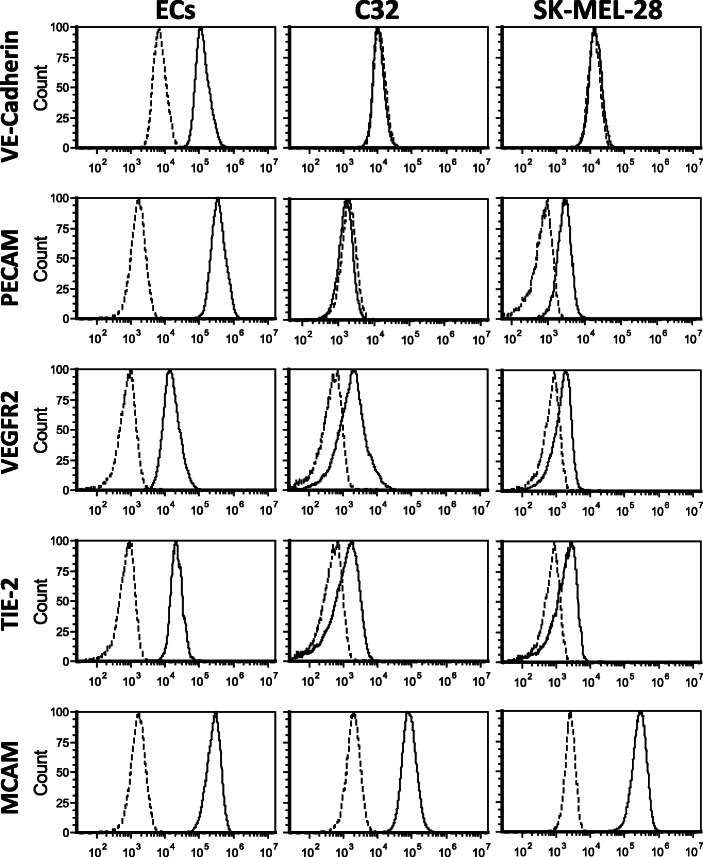
Surface expression profiling of ECs, C32 and SK-Mel-28 cells. The histograms show one representative experiment from *n* ≥ 3 replicates for each of the cell types with the dotted lines identifying isotype control stained cells and the solid lines representing cells stained for VE-cadherin, PECAM, VEGFR2, Tie-2 or MCAM

To elaborate on the potential vascular profile of the C32 and SK-Mel-28 melanoma cells, flow cytometric analysis was used to determine the expression of endothelial cell (EC) surface antigens VE-cadherin (CD144), PECAM (CD31), VEGFR2 (CD309), Tie-2 (CD202b) and MCAM (CD146). Figure 3 shows the surface expression of VE-cadherin on ECs but not on either of the melanoma cell lines. Interestingly, surface expression of PECAM was identifiable on ECs and SK-Mel-28 cells, but not the C32 cells; while VEGFR2, Tie-2 and MCAM were identified on all three cell types.

### Exogenous TSP-1 does not inhibit VM capacity of melanoma cells

**Fig. 4:**
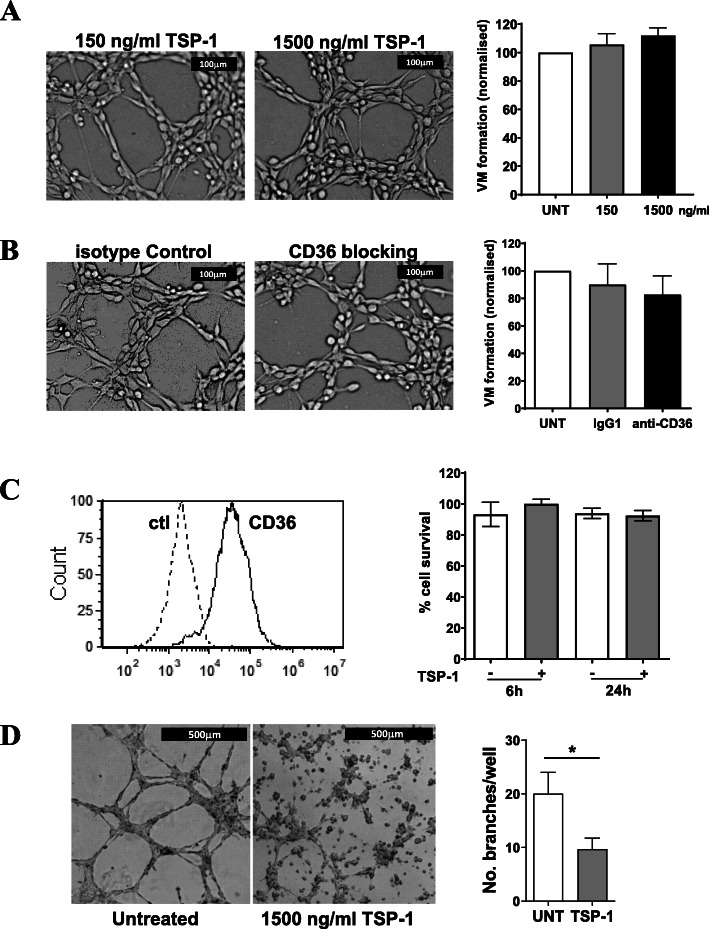
Blocking CD36:TSP-1 interactions does not inhibit VM by melanoma cells. **(A)** VM formation by C32 melanoma cells assessed following treatment with 150 and 1500 ng/ml TSP-1. Microscopy images are representative of VM with TSP-1 treatments (scale bar = 100 μm). Right bar graph, number of VM normalized to untreated (UNT) controls. Data are mean ± SEM from n = 3 experiments. **(B)** VM formation by C32 melanoma cells assessed following administration of anti-CD36 mAb (10 μg/ml) or isotype IgG1 control (10 μg/ml) prior to cell seeding for VM assay. Microscopy images are representative of VM (scale bar = 100 μm). Right bar graph, number of VM normalized to untreated (UNT) controls. Data are mean ± SEM from *n* = 3 experiments. **(C)** Left histogram, flow cytometric analysis of CD36 expression on HMVEC cells with isotype control (dotted line) and CD36 (solid line). Right bar graph, survival of HMVECs assessed without or with 1500 ng/ml TSP-1 for 6 h. Data are mean ± SEM from n = 3 experiments. **(D)** HMVEC angiogenesis without and with 1500 ng/ml TSP-1. Microscopy images are representative of EC angiogenesis (scale bar = 500 μm). Right bar graph is number of EC branches per well normalized to untreated (UNT) controls. Data are mean ± SEM from n = 3 experiments. *p < 0.05, t-test

To investigate whether TSP-1, a known inhibitor of angiogenesis [32, 33] and a well-documented ligand for CD36, could also interfere with VM formation by CD36-expressing melanoma cells. In vitro*,* we performed VM assays with the C32 melanoma cells in the presence of increasing concentrations of TSP-1. Based on the literature of circulating TSP-1 in patient plasma ranging from 245 ng/ml, in healthy individuals, to 3650 ng/ml, in cancer patients [38], we included 0, 150 and 1500 ng/ml of TSP-1 into the VM assays. Figure 4A illustrates that the VM structures were not perturbed by the increasing concentrations of TSP-1. Further support for TSP-1 not being the relevant ligand for CD36 in VM comes from our inclusion of the anti-CD36 blocking antibody (FA6–152) that specifically blocks CD36 binding to TSP-1 and collagen [39–41]. Figure 4B shows that addition of this anti-CD36 blocking antibody (10 μg/ml) did not inhibit VM formation, thus suggesting that neither TSP-1 nor CD36-engaged collagen are involved in VM formation by melanoma cancer cells.

Importantly, to confirm that our TSP-1 was indeed functional, we used the CD36 expressing human microvascular endothelial cells (HMVECs, Fig. 4C) in an angiogenesis assay [32, 33]. First, we confirmed that exposure of 1500 ng/ml of TSP-1 for up to 24 h did not compromise cell viability (Fig. 4C), prior to addition of TSP-1 into the angiogenesis assay which showed a significant reduction in angiogenesis by the HMVECs when TSP-1 was present (Fig. 4D). This differential between EC angiogenesis and cancer cell VM has not been reported previously and goes some way to describing the important differences between these two-contributing process of tumor vascularization.

### CD36 receptor mediates melanoma cell adhesion to extracellular matrix components

**Fig. 5:**
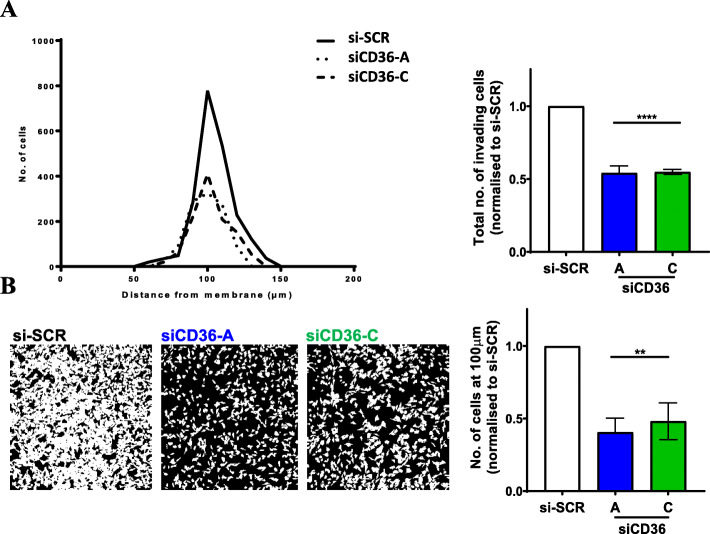
CD36 facilitates the migration of melanoma cells. **(A)** Left panel, quantitated data of the distance travelled by C32 melanoma cells towards 10% FBS with siCD36 (siRNA A or C) or siRNA control (Scrambled). Right panel, quantitated data of total number of invading cells normalized to control (si-SCR). Data are mean ± SEM from n = 3 experiments. ****p < 0.0001 vs si-SCR, one-way ANOVA. **(B)** Left confocal images are representatives taken at a distance of 100 μm from the Transwell membrane start point for si-SCR control or siCD36 (A and C). Images were captured via a 20x objective on a Zeiss LSM 700 confocal microscope. Right panel, quantitated data of the number of cells at 100 μm from the Transwell membrane start point. Data are mean ± SEM from n = 3 experiments. ** *p* < 0.01 vs si-SCR, one-way ANOVA

To further investigate the role of CD36 on VM-competent melanoma cells, we performed inverse invasion assays wherein we tested the ability of C32 melanoma cells (without or with CD36) to crawl through an extracellular matrix (Matrigel) towards a chemoattractant (10% FBS). Figure 5A shows that over 48 h, the cells travelled between 50 and 150 μm from the Transwell membrane with the majority travelling 100 μm over that time. The loss of CD36 (via siRNA knockdown) did not impact on the cancer cells being able to invade up to 150 μm. However, we observed that loss of CD36 significantly inhibited the number of C32 cells that migrated towards the FBS (Fig. 5A). This difference is best exemplified in Fig. 5B which illustrates the number of cancer cells at precisely 100 μm from the Transwell membrane start point. With CD36 knockdown, there is a 50% reduction in cancer cells at this position. This data implies that CD36 facilitates an interaction between cancer cells and the extracellular matrix, and from our results above, CD36 is unlikely to interact with TSP-1 or collagen.

### Melanoma cells utilize CD36 to bind to laminin substrata

**Fig. 6:**
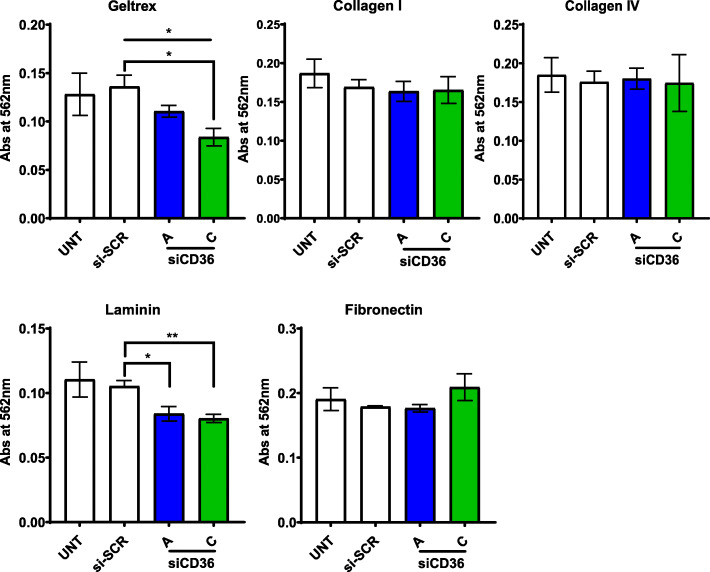
CD36 aids selective adhesion by melanoma cells to components of the ECM. Rose Bengal staining of C32 melanoma cells, without and with siCD36 knockdown (untreated (UNT), siRNA control (si-SCR), and CD36-targeting siRNA (siCD36 A and C)) following 90 min exposure to plates coated with Geltrex, Collagen I, Collagen IV, Laminin or Fibronectin. Data are mean ± SEM from n = 3 experiments. * *p* < 0.05, **p < 0.01, one-way ANOVA

To further investigate how CD36 may be contributing to cancer cell invasion through the extracellular matrix components, adhesion assays were performed on Geltrex and a selection of components contained within Geltrex known to be bound by CD36, i.e. collagen I, collagen IV and laminin. Adhesion to fibronectin was also examined as a matrix component that does not engage CD36. Figure 6 shows that following knockdown of CD36, the C32 melanoma cells demonstrated reduced binding to Geltrex; adding further support to our findings of reduced VM and invasion. While no changes in cell adhesion were observed for C32 cells exposed to collagen I, collagen IV or fibronectin, when CD36 was knocked down, reduced adhesion to laminin was observed for both CD36-targeting siRNA constructs (Fig. 6).

### Binding of melanoma cells to laminin is facilitated through integrin-α_3_

**Fig. 7:**
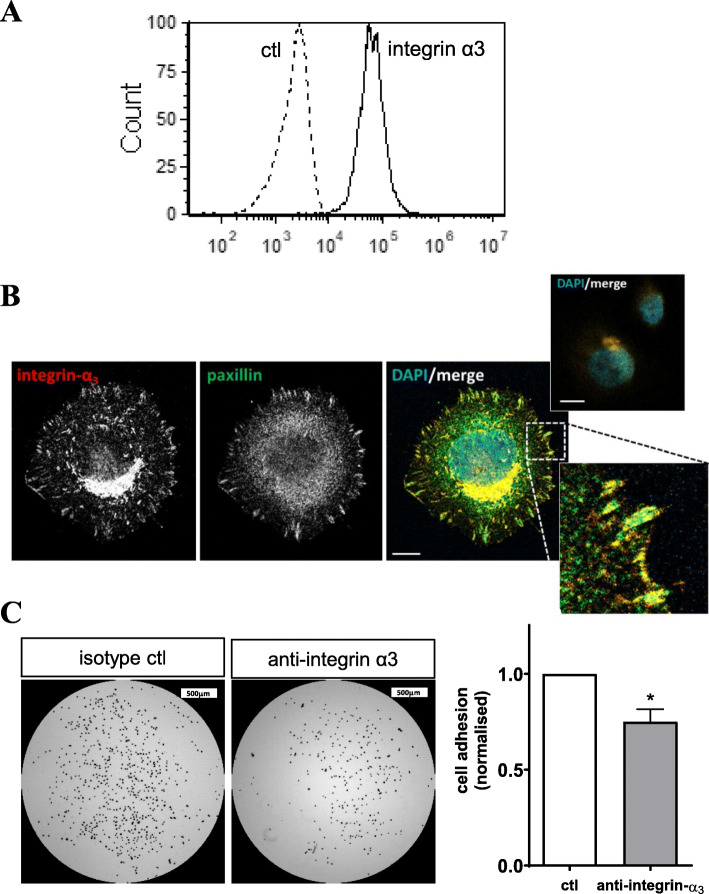
Integrin-α_3_ supports melanoma cell adhesion to laminin. **(A)** Flow cytometric analysis of integrin-α3 expression on C32 melanoma cells with isotype control (dotted line) and integrin-α3 (solid line). **(B)** Adhesion of C32 melanoma cells on to laminin coated coverslips for 60 min prior to staining for integrin-α_3_ (left panel), paxillin (middle panel) and a nuclear stain (DAPI, colored merged image). Dual stained focal contacts shown in high magnification insert (bottom right). Isotype control stains shown in the top insert. Scale bar = 10 μm. **(C)** Adhesion of C32 melanoma cells treated with an anti-integrin-α3 antibody or isotype control onto tissue culture plates coated with 50 μg/ml laminin. Microscopy images (left) illustrate the density of adherent cells on each well surface. Scale bar = 500 μm. Bar graph (right), quantification of C32 melanoma cell adhesion to laminin as assessed following treatment with a blocking anti-integrin-α3 antibody or isotype control. Results are normalized to control (ctl) wells and are mean ± SEM from n = 3 experiments. *p < 0.05, t-test

Given that Thorne *et al* previously demonstrated an association between CD36 and integrin-α_3_β_1_ on melanoma cells [42], and that integrin-α_3_, but not CD36, binds laminin, we next examined whether blocking integrin-α_3_ might inhibit the adhesion of VM-competent melanoma cells and laminin in vitro. First, flow cytometry confirmed the cell surface expression of integrin-α_3_ on the C32 melanoma cells (Fig. 7A). In support for integrin-α_3_ actively engaging with laminin, C32 cells were seeded on to laminin and immunocytochemistry identified active focal contacts at the cell periphery which contained both integrin-α_3_ and paxillin (Fig. 7B). Finally, we observed that C32 cell adhesion to laminin could be partly blocked via an anti-integrin-α_3_ blocking antibody (Fig. 7C).

## Discussion

CD36, most widely known for its role as a scavenger receptor involved in the uptake of fatty acids, is gaining interest in the field of cancer research [29, 43, 44]. Expression of CD36 has been shown to correlate with poor prognosis (disease-free survival and overall survival) in luminal A breast cancer, lung squamous cell carcinoma, bladder cancer and melanoma [29, 45], with studies primarily focusing on the role of CD36 as a receptor for oxidized low density lipoprotein (oxLDL). Our own interrogation of the TCGA-SKCM database suggests that high expression of *CD36* correlates with poor clinical outcome for patients with melanoma (albeit not significantly) and corroborates the study by Nath and Chan [45]; thus lending further weight to investigating CD36 in melanoma.

Here we reveal an unexpected role for CD36 in VM formation by melanoma cancer cells. More specifically, using the in vitro angiogenesis assay with two human melanoma VM-competent cell lines [16], we observed that knockdown of CD36 by siRNA significantly perturbed VM formation. To further examine the contribution of CD36 to VM formation, our invasion assays showed that in the absence of CD36 on melanoma cancer cells, their ability to migrate through an ECM was compromised, thus suggesting a direct interaction of CD36 with one or more components of the ECM. Interestingly, TSP-1, a known ligand of CD36 that inhibits angiogenesis by ECs [32, 46], was unable to inhibit VM formation by the cancer cells. Congruent with this, a CD36 blocking antibody (clone FA6–152) that specifically targets the epitopes for TSP-1 and collagen [39–41] also failed to prevent VM formation in our assays. Taken together, this data suggests that CD36-expressing VM-competent cancer cells may utilize CD36 differently to ECs for the formation of vascular structures; a process that is yet to be fully elucidated. Transient knockdown of CD36 in melanoma cells did not influence cell viability but did compromise the number of cells that migrated, again suggesting a role for CD36 in cell adhesion. This supports recent studies wherein CD36 proved important for the migration and invasion of breast cancer cells and cervical cancer cells in vitro [30, 47]. More specifically, these studies show that CD36 can promote the activation of the MAPK signaling pathway, upregulate protein expression of Bcl2 and cyclin D1 and engage with the TGF-β signaling for epithelial to mesenchymal transition (EMT) by cancer cells [30, 47].

**Fig. 8:**
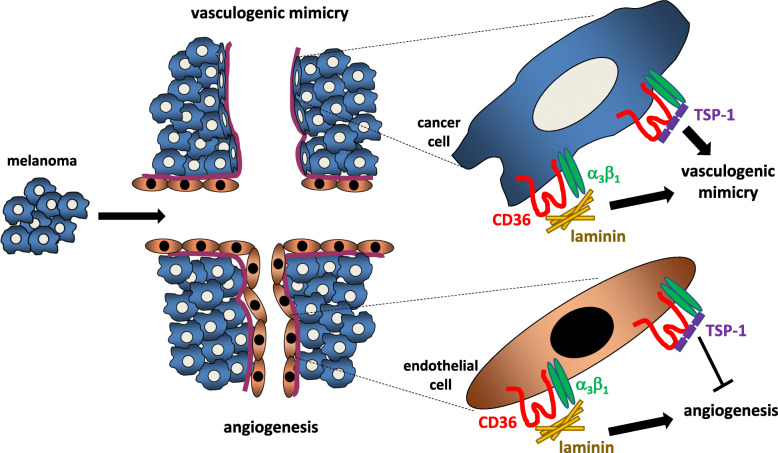
Schematic concept of CD36 in VM formation and cancer progression. Solid tumor growth is underpinned by angiogenesis and VM. We hypothesize that CD36 expression by tumor ECs and the cancer cells themselves interacts with integrin-α_3_ to promote adhesion to the tumor ECM (particularly laminin) thus facilitating tumor vascularization (i.e. angiogenesis and VM) and cancer progression. Thrombospondin (TSP-1) is a ligand for CD36 and inhibits EC-lined angiogenesis. By contrast, VM by highly malignant melanoma cells is unperturbed when TSP-1 binds CD36. This schematic was conceptualized and crafted by CSB using Microsoft PowerPoint

To further elucidate the role for CD36 on cancer cells to engage with ECM components, adhesion assays were performed on collagen I, collagen IV, laminin and fibronectin, all of which are in high abundance in the ECM of tumors [48] and all are documented ligands for CD36 [42]. Here we observed that loss of CD36 significantly reduced the ability of melanoma cells to bind to the laminin substrata while retaining full binding capacity to collagen I, collagen IV and fibronectin. The retained binding to collagen is supported by our observations of the anti-CD36 monoclonal antibody (clone FA6–152 [39–41]) failing to inhibit VM formation by the cancer cells. Our observation of CD36-knockdown melanoma cells exhibiting reduced adhesion to laminin concurs with a study by Ladanyi and colleagues who demonstrated that ovarian cancer cells utilize CD36 for adhesion to laminin [27]. A potential mechanism by which this interaction may occur is via a lateral association with integrins, specifically the laminin binding integrins α_3_β_1_ and α_6_β_1_ [49, 50] both of which have been reported to associate with CD36 in human melanoma cells for enhanced migration on ECM components [42]. Here we reveal that like CD36, blocking integrin-α_3_ on melanoma cells inhibited the binding of melanoma cells to laminin in vitro. Taken together, this study leads us to hypothesize that CD36 expression by melanoma cells modulates the function of integrins to promote shape change and migration on ECM components, thereby facilitating the formation of VM structures (Fig. 8).

Increasing literature demonstrates that overexpression of CD36 in cell lines of cervical and oral squamous cell carcinoma significantly increases their metastatic potential in vivo [29, 30]. In accordance, CD36 neutralizing antibodies have been successfully used to combat metastasis in mouse models [29]. More specifically, repeated injections of the anti-CD36 antibody (clone FA6–152) significantly attenuated the metastasis of oral squamous cell carcinoma without effect on the primary tumor [29]. Whether these anti-CD36 treated tumors contained fewer VM structures was not determined.

Our *in-silico* analysis of the TCGA database supports our hypothesis that high expression of *CD36* correlates with poor outcome for patients with melanoma. Whether CD36 could serve as a useful biomarker for personalized medicine is not known, but with clinical trials targeting CD36 for patients with metabolic disease [43, 51], an opportunity to repurpose an anti-CD36 molecule remains of interest. Notably, caution surrounding CD36 as a target to treat cancer is unsurprising given that it is a fatty acid scavenger receptor with a wide range of ligands. Indeed, lipotoxicity was observed in tumor bearing mice treated with the CD36-neutralising antibody [29]. Clearly, while the refinement of CD36-targeting antibodies requires significant attention, there is increasing interest in CD36 as a potential prognostic for patients with colon, ovarian, breast, small cell lung carcinoma and urinary bladder cancer [29, 43, 44] and melanoma. This study adds to this interest by identifying a key role for CD36 in the lateral interactions with integrin-α_3_ to promote adhesion to the ECM (particularly laminin) to facilitate VM formation, an emerging process that contributes significantly to the progression of cancer (Fig. 8).

## Conclusions

The results from this study identify CD36 as a previously unrecognized regulator of VM by melanoma cancer cells. The VM process is multifaceted and our study suggests that CD36 contributes by co-operating with adhesion molecules (such as integrin-α_3_) to engage with components of the tumor microenvironment (such as laminin). Interestingly, our results suggest that VM differs from angiogenesis in that TSP-1 does not inhibit VM formation whereas it does perturb angiogenesis by ECs. This new information contributes to our understanding that both ECs and cancer cells form vascular structures within a tumor mass via processes that are not entirely identical. These differences will become important as we work towards specifically targeting tumor vascularization to best combat cancer progression.

## Data Availability

The data analysed in this study are available in The Cancer Genome Atlas (TCGA) repository together with RNA sequencing (RNA-seqV2) and clinical (Biotab) data available from the Data Portal at http://cancergenome.nih.gov.

## Abbreviations

EC: Endothelial cell
VM: Vasculogenic mimicry
TSP-1: Thrombospondin-1
VE-cadherin: Vascular endothelial
EphA2: Ephrin receptor A2
PECAM-1: Platelet endothelial cellular adhesion molecule
PAS: Periodic acid-Schiff stain
MVECs: Microvascular ECs
FBS: Fetal bovine serum
siRNAs: small interfering RNAs
HMVEC: Human lung microvascular endothelial cells
BSA: Bovine serum albumin
ECM: Extracellular matrix
TCGA: The Cancer Genome Atlas
EMT: Epithelial to mesenchymal transition
